# The Anatomist's Instructor, and Museum Companion

**Published:** 1836-10-01

**Authors:** 


THE ANATOMIST'S INSTRUCTOR, AND MUSEUM COMPANION:—BEING PRACTICAL DIRECTIONS FOR THE FORMATION AND SUBSEQUENT MANAGEMENT OF ANATOMICAL MUSEUMS. By *Frederick John Knox*, Surgeon, Conservator of the Museum in old Surgeon's Hall. 12mo, pp. 152. Edinburgh, 1836.

IN our last number we noticed at some little length a paper by Mr. Crosse, of Norwich, on the best mode of preserving preparations in spirit. We did so, because that is very useful knowledge, and many surgeons, both in town and country, are much in want of it. We believe that few have been more anxious than ourselves to encourage the taste for pathological investigations, and to point out the indispensable necessity for becoming conversant with the facts of morbid anatomy. The Tory Doctrinaires are ever in alarm when morbid anatomy is mentioned, and always attempt, though sometimes obliquely, to throw cold water on its study. The impulse that it gives disturbs their inertia. But they have been driven from post to post, and although they have defended gallantly enough, the entrenchments of good old empirical ignorance, they have little ground left on which to fight.

It is amusing to look back on their defeats. When the diseases of the lungs were investigated by Laennec, they raised the battle cry of dogmatism, and shouted against the pathological innovator and his inutile lignum. How they derided the notion of pneumo-thorax, and the absurd idea that abscess of the lung was not a common thing. But pathological facts prevailed against prejudice, and the curious observer may now frequently discern a physician of the school that laughed at auscultation, solemnly applying the wrong end of a stethoscope to the wrong part of a chest, and profoundly recording what he *cannot* hear. Ridiculous as this may seem, it is the surest test of the triumph of truth, for it is easier to convince opposing reason, than to shame ignorance and ancient prejudice.

When the scalpel was applied to the investigation of the nature of fever, again the old party denounced the folly of the morbid anatomists. How they chuckled at the notion of follicular ulceration, how they clung to the good Hippocratic doctrine. Fever was from beginning to end a hocus-pocus thing—they could not tell, and nobody knew what; and it was a dream of pathological enthusiasts to suppose that its nature could be unravelled by dissection. But again the truths displayed by morbid anatomists prevailed, and although its sanguine advocates rather overrated what it could effect, yet no one can deny that our acquaintance with fever has been rendered infinitely more exact and philosophical than it was before the organs involved in it had been carefully and extensively examined after death.

It would be useless and impertinent to pursue these reflections, or to waste time in proving what most well-informed men now admit, that the dissection of disease has within these few years effected incalculable good for medicine, and is still continuing to effect it. The alterations of the heart and arteries—the diseases of the brain and of the spinal cord—the affections of the joints and of the urinary organs, are those in which most has been latterly accomplished. The researches on the varieties of morbid growths and malignant changes of structure, have unravelled something of the obscurity which still surrounds them; and when we look, on the one hand, at the first description of fungus hæmatodes by the older Hey, and contemplate, on the other, our present state of information with respect to these complaints, we have much reason to be satisfied with what has been done, and some to anticipate what may be done hereafter.

In large towns, where there are hospitals, and museums constructed and supported at a great expense, we are not only enabled to place ourselves on the level of the information of the day, but even to contribute our share to its advance. But the country surgeon removed from cities where these advantages may be procured, obtains with difficulty the means of extending his pathological knowledge. It is true that the journals inform him what is going on in the larger theatres of science, and many excellent publications, intended to disseminate the knowledge of pathology, reach him. But his opportunities of becoming practically conversant with changes which he sees depicted in drawings, or described in words, are few and far between, and it is a very great object that he should be qualified to make the most of them.

To do this he should know the best methods of preserving those parts, which he has had such difficulty in procuring. This is really useful knowledge, because it preserves what will continually remind him of what he might otherwise forget, and, more than that, it breeds in him a taste for researches, which cannot be pursued without giving a philosophical character to his mind. But unfortunately this necessary information is seldom acquired in the period of pupillage, and hitherto there has been no work to which the surgeon could refer for such full and such accurate directions as would be of real service to him.

It is impossible not to see how rapidly the mass of the profession is rising in its intellectual appetites. All now are anxious to know something at least of natural history, of comparative anatomy, of botany, of geology, and of many sections of science or of art, in which the merest medical smatterer half a century ago would have been deemed a Crichton. No doubt that this taste and these habits contribute to make men sciolists. But a man in our profession cannot expect, if he practises it as he should do, to be more than a sciolist in other things. To medicine his attention must be mainly directed, and to it he must consecrate the general information he possesses. Yet wherever he is, there are the means of getting specimens of natural history, and of comparative anatomy; and if a surgeon possesses the wish to collect, and is acquainted with the means of preserving them, we may safely say that there is no one, however small his means, or disadvantageous his position, who may not gradually accumulate something like a scientific museum.

To aid him in that interesting and useful occupation is the object of the little book that lies before us. Its author, Mr. Knox, a conservator of the museum in old Surgeon's Hall at Edinburgh, is qualified from experience to teach, and it shall not be our fault if "The Anatomist's Instructor" is not in the hands of most members of the profession.

It is divided into three parts. The first is appropriated to the Mode of Forming Anatomical Museums. The second is on the Modes of Preserving Diseased Structure illustrative of Pathology. The third is on the Art of Drawing, Modelling, and the Use of the Microscope.

It would, of course, be out of the question to analyse a manual. We shall therefore select those portions which seem to us to convey the information we would wish to see extensively disseminated, and recommend such of our readers as are anxious to be au niveau of the mysteries of museum-making, to purchase the work, and keep it constantly at hand.

The first chapter is on the various methods of preserving the soft textures.

*Preservation by Drying.* This is in common use. Every student knows that a very oily preparation will not dry well. Labour is generally thrown away upon it. Never put up an oily preparation wet or dry, unless it is very valuable indeed. The first thing we must do before we dry a preparation is to get the blood out of it as much as possible by maceration in water. The water should be changed at least twice daily, and as soon as it is no longer tinged with blood, the maceration should cease. But the drying itself is a thing of consequence. Let us hear Mr. Knox.

" Those preparations intended for drying will either require to be stretched out by means of pins, on a board composed of soft wood, and perfectly smooth; or, in the event of being hollow, such as the stomach, bladder, &c. they must be distended with air, if entire, or, if not admitting of being distended with air, may be stuffed with baked hair or wool. Oiled paper, such as is used for tracing, may be used, so as to prevent the preparation adhering to the board or the material made use of in stuffing." 10.

But the student must not think that if a preparation is hung up, it will therefore necessarily dry well. Frosty weather won't do, and moist weather is worse still. Hang the preparation in a draught, and a draught of dry warm air if it can be got. If it can't, we are not to despair—far from it—for, then:—

Let the part be put in a dish, " covered with proof spirits, and evaporation prevented by means of a close cover; or, if bulky, merely wrapped in a cloth moistened from time to time in alcohol. It may afterwards be finished off at any period, and it dries and looks the better by having been immersed in spirits."

This partial or complete immersion in spirits, prior to the operation of drying, increases the beauty and facilitates the desiccation of membraniform preparations vastly. It is even of very great service in putting up blood-vessel preparations, and this is the plan which, at some schools, is always adopted.

We now approach a very serious and momentous question—one on which we anticipate great doubt and difficulty. Ought preparations to be varnished? There's the rub. Our author has taken a deliberate vow against varnish. It matters not what varnish it may be, mastic, copal, or white spirit—

He'll have none on't.

" My objections to the use of varnish, I am aware, will require to be clearly and forcibly stated, as its use is at present almost universal. Whilst in charge of the Museum of the College of Surgeons, an extensive collection of vascular preparations was presented to the College by Dr. John Thomson. They had all evidently received abundance of varnish; and in consequence of the succession of coats of varnish and dust obscuring the arteries and veins, an attempt had been made to paint these. I observed, in the mean time, that these preparations had not been in the museum for many hours, when, on lifting any one of them, there invariably remained various little hillocks of a fine brownish-looking dust. I knew this to be the effect of a very active small black beetle, common in museums, and instantly gave these preparations a thorough examination. I found these beetles in thousands; they had undermined every texture, the thick coating of varnish in many parts only remaining. It is true that repeated applications of turpentine destroys all insects; but the varnish so effectually prevented the turpentine from reaching the central parts, that I despaired of completely clearing the preparations. I kept them in an apartment separate from the museum, and gave them a daily visit for many months. My mode of preserving the largest vascular preparations, however great the quantity of muscle may be, is simply to dry it, having previously cleared it as effectually as possible of blood, and soaked it well with spirits of wine. During the winter months a little mould will often make its appearance, even in the best-arranged museums; but this can be instantly removed by means of turpentine applied with a brush. In summer, air should be freely admitted to the cases containing the preparations, and turpentine freely applied. If oil should appear on any part, it may be removed by means of a little tow. I have now in my possession vascular preparations preserved in this manner ten years ago, and which look quite as well as when prepared, and, I may add, as well as any I have had an opportunity of examining. The Barclayan Museum was very rich in vascular preparations, and, having had it under my care for a length of time, I observed that all these preparations had been varnished. The insects were abundant, but not so numerous as in the collection to which I have already alluded. The greater proportion of those in the Barclayan Museum were intended to shew the number of minute branches into which arteries divide, having all the muscles nerves, &c., cut away, so that little was left for insects to pick at. Another set of vascular preparations, of very superior utility to the minutely dissected ones, had suffered prodigiously from insects, the material used in filling the vessels having mostly been composed of tallow." 12.

There is something in these observations to give us pause, and to excite a very solemn train of reflection. Is there a conservator whose conscience can acquit him of having used varnish in a reckless way? Does a preparation look shabby?—Varnish it. Is it decaying?—Varnish it. Is it the last week in September?—Varnish it for the first of October. Is the museum to be disposed of? Order a dozen pots of varnish. Poor Mr. Brookes was a glutton in varnish, and we hear that its consumption, prior to the disposal of his preparations, was frightful.* We own that, not having the fear of "small black beetles" properly before our eyes, we have ourselves infamously over-varnished. But we will reform, and we recommend our friends and readers to take counsel. Yet, as the Member for Finsbury would say, we are "free to confess," that we cannot go the lengths that Mr. Knox has gone, and eschew varnish altogether. Only look at that varnished limb. It has not been done with a mop, but lightly, gently, rapidly, and con spirito. How glossy, how elegant, how adapted for a museum! And see that other dry, crusty, sulky-looking, unvarnished thing beside it. Oh, we cannot give up varnish; but we promise, and may the Gods record our adjuration, that we never will abuse it. We intend hereafter to confine ourselves to white-spirit varnish, to put on the thinnest film imaginable, and never to employ it when the preparation is much exposed to dust. With these precautions, we think it may be very safely indulged in.

Before we quit this absorbing subject, let us say one word about cleaning varnished preparations. When they become dirty and dusty, many people, proh nefas, content themselves with laying on a fresh dab of varnish. This shews not only want of principle, but ignorance. It is, as Fouché says, "worse than a crime—it is a blunder." The fact is, that many varnished preparations may be cleaned with a brush, and a little warm soap and water. This removes the external dirt-absorbing surface of the varnish, and, after the preparation is dried, a fresh thin layer of varnish should be applied. Of

---

* The College of Surgeons have been grossly abused for not purchasing that museum. They exercised a most wise discretion, for, with many good preparations, there was a host of bad ones, and an inconceivable quantity of rubbish.

course there are many preparations, for which the employment of soap and water would be sacrilege.

Turn we to wet preparations. What a source of anxiety these are, and ever have been, to curators, it is unnecessary for us to say. A really good preparation in alkohol is an object of as much solicitude to him as her lamb was to Maria. He fondles it—watches it, but, as the Gheber's bride pathetically laments—

In spite of lead and bladders's power,
He sees his fondest hopes decay ;
And still, alas, from hour to hour,
The alkohol *will* fade away.

Will no benefactor to his species arise, to shew us how a wet preparation can be really tied up securely? No such has appeared on the horizon yet, and, for the present, we must be contented to do the best we can.*

There is not a more painful spectacle to a curator, than seeing a clumsy brute of a fellow attempting to put up a spirit preparation. It is shocking to see the creature *mauling* the delicate thing, as if he was making a dissection for lecture. What must have been Mr. Knox's disgust at the following occurrence, which he relates with becoming indignation. We admire his spirit in refusing to have any more to do with the dirty work. It is a pity he did not throw the thigh-bone at the rascal's head.

"The whole process of preserving anatomical objects in a soft state, requires a care which few persons have the slightest idea of. I remember a gentleman, who had very considerable pretensions to be ranked as a pathologist, presented to the museum a preparation of fracture in the neck of the femur. The opinions of surgeons regarding the pathology of this accident were then, as now, very various ; and the number of opportunities of observing the condition of the injured parts in a recent state being comparatively few, great care was bestowed in dissecting the specimen. By-and-by the gentleman required the preparation for his lectures, and it was, according to a regulation of the Royal College, given to him. Upon being returned to me, I found it covered with dust, hairs, &c. ; and, on inquiry, found that it had been removed from the jar, handed about the class on a dirty trencher, wiped by the common door-keeper, with the cloth with which the seats of the class-room had been *carefully* cleaned for the preceding week ! I refused to receive the preparation into the collection." 13.

The scoundrel ! But we will not trust ourselves with the expression of our feelings. The dissection of a part that is destined for spirits should be done under water, good clean water—if dissected at all on cloths, they should be fine, and very clean also—all dust and dirt must be religiously eschewed—in fact, the dissector must lay to heart good old Isaac Walton's advice for impaling a live frog—

"Treat him tenderly, as you would your brother."

The following recommendation is worth listening to.

"In dissecting delicate preparations for preservation in spirits of wine, such as the eye, the anatomy of the fœtus, and indeed all membraniform textures, it

* We spoke so fully of wet preparations in our last Number, that, in the present article, we shall simply introduce a few unconnected additions to those observations. We refer the reader to that Number.

will be necessary to have them fixed and under water; this is effected by having a few pudding-dishes, the bottoms of which you have previously covered with wax, in the manner as directed for making wax tablets,* only not oiling the bottom of the dish, when the wax will be found to adhere to the bottom. The advantages of this mode of preparation is, that after you have dissected and prepared the preparation under water, you can pour off the water and substitute alcohol, without moving the preparation, and thus harden it, and indeed fashion it to any form desired; and this form it will be found to keep when finally put up. If the parts are extremely delicate, the jar and tablet having been selected and prepared, the wax tablet may be used at once for the dissection, and the whole process thus conducted off-hand, and no farther change made, except the transfer of the tablet and preparation from the flat dissecting-dish to the glass jar." 16.

We shan't say a word on the modes of preventing the evaporation of spirit from the bottles. Do what we may, spirit *will* evaporate. It's consistent, as Square would say, with the eternal fitness of things—all we can do is to keep down evaporation as much as possible. We don't see that any thing material can be added to what we said on this subject in our last Number, and therefore we proceed to inquire how a preparation should be preserved in turpentine.

One great virtue of turpentine is, that it forms a specific against all insects, from the maggot to the "small black beetle." This is enough of itself to endear it to anatomists.

"All preparations which are to be preserved in turpentine must be first *dried;* and in our enumeration of the various more striking preparations, we shall particularly note those which are to be preserved in turpentine. We shall here confine our observation to the mode of securing the turpentine in the jar. In the Barclayan and Bell Collections, when put under my charge, a very considerable number of preparations were preserved in turpentine; but, particularly with regard to the former, the attempt was an entire failure, in consequence of the turpentine constantly trickling down the outside of the jar, and, when partially dry and mixed with dust, becoming a kind of paint, rendering the preparation in general invisible, and the jar itself perfectly intangible. Those thus prepared in the Bell Collection were not just so bad; and this, I observed, was owing to the superior form of the neck of the jar, and the extreme care which had been taken in inclosing the preparation. The preparations, we have remarked, are to be first dried; and therefore most of them will be found not to require *suspension,* provided the jar is properly suited in size to the preparation. Should a means of suspension be required, a portion of wood must be used, as the threads must on no account pass over the rim of the jar. These preparations should always be put up during Summer, or in a room properly heated, and the jar filled quite to the brim. A single layer of *very soft (but not putrid)* bullock's

---

* "Wax-tablets, made by melting common but good bees'-wax over a slow fire, and carefully mixing any colour, such as vermillion, king's-yellow, imperial blue, &c., and whilst in a fluid state pouring it out on a flat dish, previously slightly oiled. Upon cooling, it may be removed from the dish, and cut into tablets, so as to suit the jar fitted for the display of the preparation; delicate membraniform parts can be stuck on these tablets by means of bristles or common pins, and a deal of their anatomy displayed. These wax-tablets ought to be at least 3-16ths of an inch in thickness, and instead of mixing a colour the wax may be simply dyed."

bladder is then to be stretched over the top, and neatly secured with two or three circles of fine twine, particular care being taken that no air is allowed to remain between the bladder and the neck of the jar. The bladder should then be trimmed away, but not too closely, and must be so soft as to adhere perfectly to the jar.* Nothing more is required; the bladder very soon dries, imbibing a quantity of the turpentine, so as to become semi-transparent; and, if proper attention has been paid to the softness, and at the same time *goodness*, of the bladder, such as being quite sound and free from fat, there will be no trickling down the sides of the jar; and, indeed, so far as I can observe (from some preparations in my possession, and many in the Museum of the Royal College of Surgeons, and which have been standing there thus prepared by myself, for six or eight years), no change whatever on the turpentine." 21.

The second chapter is on injecting and injections. It is just as ridiculous to think of making a good injection by rule, as to attempt to manufacture a cook out of Mrs. Glasse's receipts. In fact an injector, like a poet, nascitur non fit. It would be tedious to enumerate the qualities necessary to make a first-rate artiste in this way. An eagle eye, the most unruffled coolness, and genius, natural, decided genius, are requisite. See an ordinary man with an injecting-syringe in his hand. All is fuss, trepidation, confusion, and disaster. Ten to one, he squirts the boiling injection into your eye, if you are fool-hardy enough to go near him.

All that we shall do is to subjoin the formulæ for hot and cold injections, and recommend our young friends to go into the injecting-room at their several schools, and see how the whole business is conducted. Practice only can teach them to inject. The formulæ are the following:

### Size, or Minute Injection.†

Take of the best glue one pound, break it into small pieces, and into an earthen pot, calculated to contain considerably more than the actual quantity of injection wanted, put the glue, and, pour over it three pints of cold water; let it stand for twenty-four hours; then liquefy the whole by means of a gentle heat, and strain it through a flannel cloth. The colouring ingredient may be then slowly added, stirring all the time with a wooden pestle, so that no knots may be formed.

*Red.*
Size, one pint.
Vermilion, three ounces and a half.

*Yellow.*
Size, one pint.
King's yellow, two ounces and a half.

*Blue.*
Size, one pint.
Fine blue smalt, six ounces.

---

* "The very singular fact of one layer of bladder preventing the evaporation of turpentine was discovered by me by pure accident."

† This is very minute injection. The subject of the part must be previously immersed for an hour or two in water at a blood heat, and it must be kept under water during the injection. The size injection does not *set* quickly, so that the operator may take his time. To fix it, either a *little* wax injection may be thrown into the vessels when pretty full of size, or what is more convenient, cold water may be run on the injected parts. The preparations are usually preserved in alcohol or turpentine.

### VARNISH, OR FINE INJECTION.

*Red.*

Spirit varnish, eight ozs. } Measure.
Turpentine varnish, one oz. }
Vermilion, one oz.

*Yellow.*

Spirit varnish, eight ozs. } Measure.
Turpentine varnish, one oz. }
King's yellow, one ounce and quarter.

*Dark Blue.*

Spirit varnish, eight ounces. } Measure.
Turpentine varnish, one ounce. }
Blue verditer, four ounces.

"This injection *sets* very rapidly, and requires a certain degree of heat to keep it fluid. Of course the subject also requires to be heated. Should more be mixed than is required, it will keep very well, and can be restored to its fluid state by a gentle heat, with the addition now and then of a little turpentine varnish; but I must admit that the injection is most successful when the ingredients are exactly in the proportions given above, and newly made. It fills extremely minute vessels, and is intended for parts which are to be dried, such as the *principal* arteries of the body, to shew their anatomical and surgical relations. The colouring ingredients should be previously mixed in a small quantity of the varnish with a wooden pestle, and then slowly added to the remainder, so that the whole may be perfectly liquefied and smooth. The above injection will become soft, if exposed to great heat, and would scarcely support its own weight in a preparation very minutely dissected." 30.

### WAX, OR COARSE INJECTION.

*Red.*

Bees' wax, sixteen ounces.
Resin of best quality, eight ounces.
Turpentine varnish, six ozs. Measure.
Vermilion, three ounces.

*Yellow.*

Bees' wax, sixteen ounces.
Resin, eight ounces.
Turpentine varnish, six ounces.
King's yellow, two ounces and a half.

*Dark Blue.*

Bees' wax, sixteen ounces.
Resin, eight ounces.
Turpentine varnish, six ounces.
Blue verditer, ten ounces and a half.

All the preceding injections require the subject to be heated. The following is a form of Cold Injection, which entails no such troublesome necessity.

### PAINT, OR COLD INJECTION.

The colour may be according to the taste of the anatomist, but *white* is the easiest procured, and is in every respect the best suited for this injection. All colour-shops keep the white-lead prepared, *i. e.* ready ground and mixed with oil. To a small quantity of this add turpentine varnish, until of the consistence of paint, as used by painters; a very small quantity of turpentine may be added just before injecting, and, lastly, a little water sprinkled on the surface. It is then to be injected. It will generally require two or three hours to harden.

This injection can be employed only for preparations that are intended to be dried.

We have given these formulæ, because there is much difficulty in determining the exact proportions for injection. Either it is too hard or too soft, too brittle, or too easily melted. Hot wax injections are the most troublesome things in the world to manage. Such of our readers as like may try Mr. Knox's recipes. Mercurial injections need not detain us.

Corroded preparations look vastly well, and are indispensable ornaments in a museum. But they do not last long, and they are difficult to make. After the organ to be corroded is injected with wax, it should be immersed in a solution of three parts of muriatic acid, and one part of water. Mr. Knox assures us that six weeks' or two months' immersion will reduce all the soft parts to a pulpy mass, which may be easily washed away by a gentle stream of water. Mr. Knox has been more fortunate than we have been, for we have had kidneys and livers in the solution for months without being able to get the soft parts off, while, in other instances, simple maceration in water has done the business in half the time. We cannot help thinking there is much uncertainty about the action of diluted muriatic acid on the tissues placed in it.

Hardening of the brain is useful, in order that it may be dissected. Every body knows that this may be done with spirits of wine, but every body knows, also, that spirits of wine are dear.* The oxymuriate of mercury hardens the brain, and answers tolerably well. Mr. Swan has made great use of this solution.

---

* By the bye, we may say a word or two on the preservation of the brain or of other parts in spirits. Many persons imagine, that if a brain is put into spirit, that is all that need be done. This is a delusion. The membranes should first be carefully dissected off, wholly or in part. The brain should then be placed in just sufficient spirit to cover it. On the next or the following day, the spirit should be changed, for it is apt to become putrid and weak before the interior of the mass of brain is reached by it. Many is the brain, kept in one spirit, which we have seen putrid in the centre. Perhaps the spirit may require to be changed once more, when the brain is tolerably saturated, and, in a close vessel, a *very small* quantity of alkohol will continue to preserve it for an almost indefinite time.

Latterly, it has been much the practice to preserve limbs for dissection in the London schools, by means of spirit. It would be ruinous to immerse a whole limb in spirit. The method adopted is this. A long tin vessel is procured, with a groove in it, which receives a tin cover. The groove may be filled with water. The limb is placed in the tin, and a small quantity of spirit is poured on it. The cover being applied, the vapour of the alkohol preserves the limb, or indeed any preparations, which require to be frequently taken out and looked at.

For this plan to succeed perfectly, the following precautions are necessary.

*First,* The spirit must be frequently changed at first, until the limb is thoroughly saturated.

*Secondly,* The limb must be constantly looked at, and the spirit, as it evaporates, must be renewed. The best way is to keep cloths, wetted with spirit, round the limb.

*Thirdly,* The tin itself must be occasionally inspected, as rust is apt to end in holes in its bottom. Zinc vessels answer better than tin, but are more expensive.

A great many preparations for demonstration, or even for ordinary preservation may be kept in bottles, in which there is only a small quantity of spirit. The vapour is sufficient to keep them moist, at all events for a time.

"Oxymuriate of mercury, one ounce,
Muriate of ammonia, thirty-five grains,
Water, one pint;
the mercury and ammonia to be rubbed together in a mortar, and the water added gradually until the solution is complete. The part under preparation (a brain, for instance) will require a fortnight's immersion, but may be completed in a shorter period by substituting alcohol for the water. Should a white precipitate be thrown down during the immersion of the preparation, it must be removed by allowing a stream of water to flow over it; and this must always be done previous to finally drying, as, should any of the solution remain on the surface, crystals will be formed in abundance, which cannot be removed after being dried. Abundance of varnish and paint must follow the use of this solution, as all textures are reduced by it to one uniform colour." 39.

Mr. Knox has a sacred horror of paint, and red arterial injections. He thinks they are unnatural and dangerous. Now really we do conceive that he is rather hard on preparations, on the *mummies*, as he calls them. No doubt, if a surgeon learnt anatomy from them, he could be guilty of any thing. But they are useful things to teach from, and when anatomy has once been properly acquired on the dead body, they are very capital refreshers to the memory. We don't intend to be bullied out of our blood-vessel preparations. What would a museum be without them? Leather and prunella. Mr. Knox is very hard upon us. He first takes away our varnish—denounces paint—and abuses injected preparations of the blood-vessels. Like the people in Newgate Street, under the frightful decrease of executions, we are "losing all our amusements."

Mr. Knox is a prince of curators, but we don't think he is much of a surgeon. His first object ought to be to stick to his last. The following reasoning is very much of the post hoc ergo propter hoc kind—and smacks of Tenterden Steeple and the Goodwin Sands.

"I recollect of a practitioner in the town of Dunbar, where hernia is as common as in other places, stating that, in thirty years' practice, although called upon for his aid very frequently in cases of strangulated hernia, he had *never* once operated, and *never* had lost a single patient, having always been successful in reducing the hernia by means of the taxis. Within these few weeks, I have seen two cases operated on for strangulated hernia, and both terminated fatally, and, I must say, that the impression made on my mind by the death of these two, apparently healthy, women, is most unfavourable to the *cutting* surgeon." 40.

The surgeon may retort, that Mr. Knox is more cutting than he was. But the whole is a mere matter of moonshine, and we need not say any more about it.

### THE SKELETON.

Now, if there is one thing more frequently attempted, and less frequently successful than another, it is *cleaning* bones. It is quite disgusting to look on the nasty things that some people, even some teachers, unblushingly poke under your nose. The yellow, greasy, stinking abominations are handed about with the utmost effrontery. Well, then, how are bones to be cleaned? Some people say they are to be put on the sea-shore, to which there are two material objections—for, first, the sea-shore is not always to be got; and, secondly, if it were, it would not do:—Who is to look after the bones on the sea-shore? answer us that, Master Brook. Besides, if the sea were obliging enough to come to town, and if the bones were able to look after themselves, they would turn out brown, dirty-looking things at last. In short, bleaching bones on the sea-shore sounds very fine and romantic, and some foolish fellow having once started the idea, many other silly fellows have repeated it.

If the sea-shore is not to be procured, the sun is, at least, in this climate, for two or three months in the year. Put the bones in the sun, is a common piece of advice. Now it is the nature of bones to be greasy, on account of a certain substance called marrow—and it is the nature of grease to melt, when under the influence of heat. In consequence of the operation of this law of grease, bones put in the sun become most delectably oily, and the Gheber who has had his bones upon the leads for a month or two, finds to his delight, that, what with dirt, the influence of the atmosphere, and grease, they are scarcely worth the trouble of carrying down stairs. Let the bone-cleaner beware of the sun. Again then we say how are they to be cleaned? We cordially agree with our indefatigable author, in the following description of the process. He is a man after our own heart.

Catch your hare, says Mrs. Glass, before you skin him. Get your subject before you macerate him. But *every* subject won't do. No prudent person would take an old man, or a fat woman. Look to the grease, for there's the rub, and a rub very often to no purpose. The patient should be between twenty and forty, and some lingering disease must have caused his death, for then the marrow will have been absorbed.

"The macerating tub should be a barrel, fitted with a close secure cover, and capable of containing three or four gallons of water more than what completely covers the subject. The anatomist will take especial care to remove few or none of the muscles, particularly not to attempt, on any account, to expose the bones. For the first week the water will be tinged with blood, and ought to be completely changed every day. The muscles will now assume a soft bleached appearance, and the water will be but slightly tinged. The barrel should now be filled, for the last time, with pure soft water, and then closed. Supposing the situation to be a back area or cellar, the preparation had better not be looked at for six weeks or two months, The process, if to be completed in Great Britain, in the open air, and without the assistance of any artificial heat, will only succeed during the months of June, July, and August. Should the effect of putrefaction not be complete in six weeks, no effort will ultimately produce an unexceptionable skeleton; for if continued after this time, adipocere will to a certainty be deposited, and from the commencement of the appearance of this deposit, the bones themselves break up and rot." 43.

The bones being taken out of the macerating tub, and carefully placed in vessels filled with clean water, they should then be washed with a nail-brush and soap and water, and put into a very dilute solution of the liquor potassæ or ammonia, in which they should remain for a week or ten days. A current of water should then be allowed to play over them for half an hour, when they are to be placed in a clean tray, carefully wiped with a linen cloth, and allowed to dry slowly, by being placed in a current of air, but *excluded from the sun's rays.* They ought to be turned over repeatedly whilst drying, and at each time wiped with a linen cloth. Should the slightest appearance of oil be observed, the strength of the potass solution must be increased, the water renewed, and, if nothing else will do, an opening made at each end of the long bones, and water injected repeatedly through them.

Mr. Knox does not appear to us to be sufficiently alive to the merits of the chloride of soda or of lime. It is indispensable for making bones look well, and it tends also to diminish their greasiness. If skilfully managed it scarcely acts on them, and even if they do afterwards crumble a little sooner, the disadvantage is amply compensated by the whiteness, cleanliness, and freedom from bad smell that it procures for them. The plan we adopt for cleaning bones is this. We take care not to scrape the bones at first, but we are not so very solicitous about preserving *much* of the soft parts. The water is changed regularly till there is no more blood. Then the bones are locked up in unchanged water, and putrefaction is allowed to go on undisturbed. At the end of a month or two we look at the bones; if they are ready, so much the better: if not, we put them back in the putrid water. So soon as they are ready, they are taken out, washed, soaked in a solution of potass, and again washed, as Mr. Knox has directed. Then they are transferred to a weak solution of the chloride of lime, in which they are allowed to remain, until by twice or three times daily trying a cancellous portion with the nail, we find that softening is impending. The bone must then be taken out immediately, and dried, *in* a draught, and *out* of the sun. They usually become beautifully white, and free from any disagreeable odour.

Bones may be always re-cleaned by soap and water and a little chloride. If they become oily, Mr. Knox suggests that they should be boiled in a solution of pearlashes and water for an hour or so, then brushed with soap and water, and dried.

Every surgeon and physician should have a set of bones, and it is a great disgrace if he does not know how to prepare them.

The fourth chapter is appropriated to sections of the cranium, and modes of displaying the structure of bone. The fifth is on the mode of articulating the skeleton. And, the sixth is on natural skeletons.

The two first are not adapted for notice here, and we must refer the reader to the book itself for information on the points which they embrace. Our object is only to direct attention to matters which should be generally known, and are generally practicable.

Natural skeletons are often made, and should be made oftener. Every surgeon ought to have natural skeletons of the fœtus at different periods of growth. Then there are natural skeletons of birds, fishes, small animals of many sorts, that may be prepared with a little trouble, and will repay the leisure time expended on them. Our readers must know how to get up natural skeletons. Let us bring Mr. Knox again on the tapis.

" Previous to the fourth week of the fœtal existence, the skeleton of the fœtus is almost entirely cartilaginous. After this period, however, daily changes take place, and the examination of the skeleton is extremely interesting and instructive. To make these skeletons, great care in every step of the procedure is necessary, and a very considerable knowledge of anatomy is also essential. The instruments required are, a pair of good and delicate forceps, a scalpel short in the blade, and extremely sharp, and a pair of scissars, also short in the blades, with the joint perfectly easy. The fœtus should be fresh, at all events if the anatomist has not had much previous experience. The fœtus being placed on a perfectly clean board, the anatomist will make an incision along the mesial plane of the body, through the integuments merely, and he will proceed to remove entirely the integuments from the whole surface. The abdomen should then be opened, and the viscera removed. The muscles attached to the base of the lower jaw should then be carefully removed from that bone, and the tongue thus pulled towards the chest. The connexion of the hyoid to the styloid processes of the temporal divided, and the gullet and trachea, with the bloodvessels, dissected off *en masse* from the anterior surface of the neck. The sterno-mastoideus, sterno-hyoid, and sterno-thyroid muscles, being carefully removed from their inferior attachments, the anatomist may cautiously remove the diaphragm from all its attachments, and, getting hold of the apex of the tongue with a delicate hook, bring the entire thoracic viscera through the inferior aperture of the thorax. These important viscera will thus be procured in a fit state for examination, and the thorax will be entire. The muscles must then be removed with the same care as if the anatomist was examining each. The forceps being in constant use, and the scalpel and scissars alternately, every portion, when detached, should be immediately deposited on a slip of paper, so that no debris shall remain about the subject. This process must be continued until the whole muscles are completely removed. The periosteum covering all the bones must on no account be touched. With this membrane present and entire, the skeleton will be found of considerable strength, so that the extremities of the long bones may be reached, (injuring the ligaments as little as possible,) and by means of a *suitable* gimlet perforated. A wire must also be passed along the whole length of the spinal canal, and also into the interior of the cranium, so as to break down the central organs of the nervous system. The skeleton being now placed in pure water, the long bones and the cranium, including the spinal canal, is, by means of a small oyster syringe, to be most perfectly washed out. The preparation must then be placed in an ample dish full of pure water, which must be changed repeatedly whilst it continues to be tinged; and even a brush may, if used very delicately, be resorted to. A few drops of the aqua potassæ, or a little soda, may be added to the water.

The drying of this preparation must be conducted with care. If very small, it will, of course, when dry, be put into a glass jar, and should be placed extended on a board, using pins to keep it in a proper form, and prevent its contracting tendency, which is very great, in consequence of the parts in a cartilaginous state drying up almost to nothing. The ribs will require to be kept distended by means of portions of Bristol board, and the head should be filled with barley, or small dried peas, which will be easily shaken out when the preparation is dry. The preparation should be watched whilst drying, so that any error in the first adjustment may be corrected. No varnish should be used, but the skeleton, as soon as perfectly dry, put into a glass jar, and covered in with a single layer of well macerated ox-bladder." 68.

The natural skeleton of the fœtus may be preserved in spirits of wine, and no museum should be without some preserved in this way. The cartilage then does not shrink, and a more accurate notion is conveyed of the real characters of the skeleton. Preparations of the spine preserved in this manner look extremely well.

The skeletons of most small animals should be natural ones, or, at all events, should be partly natural. The great point to be observed in making all these preparations is to remove the soft parts very carefully and very systematically in the first instance, leaving the periosteum, and the attachments of the ligaments. The curator is not to tell a pupil, who is to tell a porter to dig and scrape away right and left, tearing up bone, cartilage, and ligament, with his clumsy fists. No conscientious curator will do this.

Beware how you meddle with the periosteum at first. After the necessary dissection, the animal must be put in water, and this should be changed twice daily till all tinge of blood has disappeared. Each day a little dissection should be done, and some progress made towards displaying the ligaments properly. When these are made out, the periosteum may be removed, to within a certain distance from the joint. The head should be cleaned separately. Mr. Knox recommends each extremity of the bones being perforated, without injuring the ligaments, and a wire, as large as can be conveniently introduced, passed completely through. This perforation had better be made by means of a small useful drill, thereby avoiding the risk of splitting up the bones, or the chance of the gimlet starting off the extremity of the bone, and passing through the hand or finger of the anatomist; besides, it is of importance to have a large orifice, and the drill will effect this with the greatest safety, both to the skeleton and the operator. Water is now to be injected into the interior of these bones, and the wire passed repeatedly through them until the medulla is completely washed out, and the bone becomes semitransparent. This must be done with all the long bones, otherwise upon being dried, they in general contain so much oil as to become nearly black and extremely filthy, and it will be recollected that the omission will not be easily remedied afterwards.

Maceration in clean water and dissection go hand in hand, until the joints are properly cleaned. Then the skeleton is immersed for a few days in a very dilute solution of the liquor potassæ, a careful examination of it being daily made, and if the solution becomes dirty and turbid, fresh being made. The next step is to put the specimen in a very dilute solution of the chloride of lime. A day or two of immersion in this will be sufficient, and night and morning the skeleton should be inspected. If the ligaments appear to be dissolving, or a sort of gelatinous pulp is seen on them, eschew the chloride, and set about drying as quickly as may be.

The position of the skeleton is a matter of no little consequence, but for directions and details on this head we must tell our readers to look at the book.

The skeletons of birds require, almost without exception, to be natural ones. Though many of the bones contain air, instead of medulla, yet it is a good general rule to drill, and wash out all the long bones until they become transparent. The form of the pedestal should correspond with the nature of the bird, and taste is required in arranging the attitude, and so forth. For more particular directions with respect to the skeletons of birds, and especially to those of fish, we must refer to the book itself. It will be in the hands of every body who intends to make preparations, whether in anatomy or natural history. One remark, however, may be made on the preservation of the skeleton of fishes. Alkohol has a powerful effect upon their flesh, and Mr. Knox has succeeded in obtaining the most perfect specimens, when they have been for a length of time immersed in strong spirits. With a pair of common dissecting-forceps in each hand, the flesh may be picked off, leaving the bones connected by their natural ligaments uninjured.

We now take our leave of the skeleton. The second part of the manual is occupied with morbid anatomy—with the modes of preserving specimens of altered structure.

We like Mr. Knox for his thorough-going detestation of whatever baulks a good preparation. It is sickening to see the namby-pamby listlessness of the present day. People are liberalising themselves out of everything like enthusiasm. Dandy curators talk of the opera, and are afraid of a bad smell, save the mark, because the ladies wonder what it can be which makes the room so unpleasant. Give us a right down hearty anatomist, who loves a stench for a stench's sake, and makes no bones about grubbing with the worst bones. He is the true artiste—the Ude of the dissecting-room. We fraternise, as the revolutionary people say, with Mr. Knox.

What is truth ? said Pontius Pilate. It is not to be found with the scrubs of a museum—that is certain. The articulators, and all that class, are almost as great liars as grooms, which is saying not a little. It isn't merely that they tell lies—that's a trifle. They are practical, universal liars—what they do is a lie. A scoundrel of this stamp will—

"Unhesitatingly proceed to substitute the bones of other animals, when those of the skeleton which he may be putting up may happen to be unsound, or destroyed by using too great liberties with it in articulating. The non-professional, I find, are exceedingly anxious to have every thing *complete* about a skeleton, and they carry this so far as to supply with a formidable set of teeth jaws which *never* carried teeth of any kind. They will even put in a piece of wood, rather than allow any deficiency to appear. What is worse than all this, they attempt to *improve* nature, and do not hesitate to introduce an entire phalanx, or even a new toe, into the foot of an animal, merely because the foot seems *to them* ill formed. The skeleton of the ostrich, which Cuvier has represented in one of his works, must have been the handiwork of some such mechanist. It is a fact, that instances have occurred, where, because the cervical vertebræ of a bird proved troublesome on account of their number, three of four of them were unhesitatingly thrown away by the person employed to put up the specimen, and the skeletons declared to be greatly improved, in consequence of the shortened neck." 86.

Is not this infamous ? Sometimes we see the most ludicrous ignorance exhibited. In a museum which Mr. Knox lately visited, in preparing the skeleton of a rickety person (the lower jaw-bone of which had been affected with an interesting and rare morbid condition, leading to the absorption of the alveoli,) he observed the mouth had been supplied with a very complete set of teeth, and the diseased state of the alveoli very dexterously repaired with putty ! This is pretty well.

Mr. Knox recollects seeing a preparation "libelled,* 'case of fracture of the acromion process of the scapula,' which, when examined, proved to be simply the acromion process remaining as a separate portion of bone up to the adult period of life !" This is tolerably gross.

### *Preparation of diseased Bones.*

Diseased bones usually require either to be preserved in spirits of wine or to be prepared by maceration. If the morbid parts come into the hands of the anatomist during the progress of the disease, as in cases of caries, necrosis, osteo-sarcoma, &c., an examination should of course be made of

* "Libelled" is the word used by Mr. Knox. The wag ! Any body else would have said labelled.

them, in reference to the state of the soft parts, so that the morbid structure may be preserved as a soft preparation; should the bones *only* be deemed worthy of preservation, then maceration must be had recourse to. As the unfortunate patients subject to such diseases are generally extremely emaciated, there is less risk of adipocere being deposited in the progress of maceration; but we cannot too strongly urge upon the anatomist the fact, that the more expeditiously the process of maceration is completed, the better in all cases. With diseased bones, however, it will be recollected that the destruction of the soft parts must be *complete* before the bone is removed from the macerating dish, so that if looked at prematurely, and found not cleaned, the preparation must just be returned to the *same water,* and, if possible, brought into a warmer temperature. When all the soft parts are evidently reduced to a pulp, a gentle stream of pure water must be allowed to play over the preparation until every thing is washed away. The preparation is then to be put in a weak solution of the aqua potassæ for a day or two, and finally dried. It is essential to put every thing of this kind immediately into a glass jar, as dust (which every where abounds) completely destroys them, and it will be found that they can be *touched* by few, perhaps only with safety by the person who has prepared them. Such are the directions of Mr. Knox for preserving diseased bones. He makes one or two additional remarks worth noticing.

Caries of the spine, if recent, should always be carefully dissected, and preserved dry or in spirits of wine; for the vertebræ, if allowed to be for months in water, assume a false appearance of caries. In scrofulous caries of the bones, portions of the osseous texture often die, and, being loose, are in danger of floating away with the water, whilst the preparation is washed. Great care is, therefore, necessary.

Bones in the state of mollities ossium, if it is recent, should be cleaned in the manner directed for the preparation of the natural skeleton.

"The following pathological conditions of bones are generally preserved as *dry* preparations.

A. Fracture, if united, and of old standing.—Rickets.—Wasting of bone.—Inflammation (if the effects are extensive.)—Abscess.—Caries.—Necrosis, if of long standing.—Interstitial absorption.—Interstitial deposition.—Exostosis.—Spina ventosa.—Osteo-sarcoma.—Ivory change.—Skeleton in cases of hydrocephalus.

B. *Diseased conditions of bone preserved as wet preparations.* Fractures, if recent.—Inflammation, if recent. The preparation should also be injected.—Necrosis, if recent: inject.—Medullary sarcoma.—Cancer.—Fungus hæmatodes.—Mollities ossium, or as a natural skeleton.—Appearance of bones after amputation." 91.

Mr. Knox does not allude to the use of the chlorides for the bleaching of diseased bones. Yet it is certainly of the first consequence to be acquainted with the manner of employing them. It greatly enhances the beauty of the specimen, and may be managed so as not to injure it.

Mr. Knox chuckles vastly whenever the opportunity presents itself, for shewing that he is not quite ignorant of other things besides preparation making. He is a dentist of no small calibre. Cartwright is not fit to hold a candle to him. Who will say, after the anecdote we are about to copy, that our author is not a man learned in teeth?

" I have stated that I am of opinion that the public should be put right with regard to the erroneous belief that no one knows any thing about the teeth but the dentist, and I shall relate the following case in point.—A young woman, aged 15, applied to me for an affection of the teeth, occupying the right side of both the upper and lower jaw. The patient stated that she had suffered severely from toothach for a length of time, and that the teeth on the right side appeared all diseased. Her cheek was a good deal swollen, and her general health evidently not very good. I looked into the mouth, and found the mucous membrane present a remarkable white cartilaginous appearance; small portions only of the teeth were seen projecting beyond the gum, and these portions of teeth were of a deep yellow colour. I found I could remove large portions with my fingers, and I inquired if the patient had ever used any means for the cure of her toothach. She said that she had repeatedly applied a fluid by means of a piece of linen rag, and not being able to fix on any particular tooth, had applied it generally to the whole series on that side of the mouth. The patient had none of the fluid left, but stated that she had purchased it at a dear rate from an apothecary shop: That it destroyed any part of her clothes, and even the skin when falling on them by accident; and that when put into her mouth, it produced a hissing noise similar to a heated iron when plunged into water: That it at first relieved her of pain, by rendering the entire side of the mouth void of all sensation, but finding it so troublesome to use, she had given it up for some time. My remarks on this case are as follows:—The patient had laboured under a neuralgic affection of those branches of the 5th pair of nerves which go to the teeth. The teeth had evidently been free from blemish, and I think those which were sound, were the best I ever saw. The remedy sold her had been some powerful acid; the enamel was evidently entirely destroyed, and as the ivory part must, at the patient's age, be in an active state, the result may in all probability be some frightful tumour." 92.

Mr. Knox is generally happy in his illustrations, and this hit at the dentists is very good. Yet they may be so blind as to retort, that the case proves for them, not against them—the young woman having got the acid at an *apothecary's* shop. Some people, indeed, might think that the case shews nothing in any way, except that the young woman was a very great fool. Even Mr. Knox's consummate acquaintance with dental surgery might be cavilled at, inasmuch as it solely consisted in looking into the young woman's mouth, and finding that the teeth were destroyed. The probability of a frightful tumor following will, perhaps, be questioned by some.

One would think from Mr. Knox's book that surgery was at a very low ebb in Edinburgh. Setting aside the gross instances of pathological ignorance that he introduces, he informs us that "many persons, from a supposition that the teeth are foreign bodies, and implanted in the jaws after the manner of a nail in a board, use the most unjustifiable liberties in scarifying the teeth, not only of children, but adults." And a little farther on, he distinctly asserts that "the treatment of dislocations and diseased joints is still in the hands of the empiric." We suppose he would not say so, if it was not so. In England diseased joints are undoubtedly as little in the hands of the empiric, as any class of affections whatever; indeed, if any description of case is withdrawn from the clutches of quacks it is this. But Mr. Knox goes on to observe that "diseases of synovial membranes are admitted by all surgeons to be most important, from their extreme danger to the patient. Little is *certainly* known of their pathology; and until more is known, their treatment must want precision." We suppose that Mr. Knox never heard of one Mr. Brodie, who has been made a Baronet, and is familiarly called Sir Benjamin. Yet we assure him that such a personage has not only lived, but is actually living, and has written a book, which some people think a good one, on these very diseases of the joints. Nay, he has gone so far as to point out the tissues in which particular affections originate, and to which they extend. Mr. Knox, in his ardour for natural history, has perhaps been imitating the ostrich, a bird which, they say, puts its head in a hole, and concludes that every thing is as dark as that.

"It appears to me (he proceeds), that the principal object to be kept in view by the anatomist in his researches after the true pathology of affections of the joints, is to ascertain in what *texture* the disease has commenced, and a few carefully recorded cases, followed by equally judiciously conducted dissections, will no doubt lead to a correct line of treatment in these at present fatal affections." 95.

Passing over a good many subjects, on which we see nothing to detain us, we pause to insert the following hint with regard to the preparation of nævus maternus.

"The only mode which I can devise of preserving the tumour called *nævus maternus* (and which in truth is simply a congeries of veins and arteries communicating with each other) is, first to wash out the blood by means of a syringe, and repeatedly changing the water in which the specimen must be first put. A quantity of spirits of wine should then be injected into the tumour, and the whole immersed in the spirit. After hardening, a section should be made, and finally put up as a wet preparation. In all cetaceous animals, we find a prodigious quantity of this remarkable structure as a natural and perfectly organized texture in them, although constituting a disease in the human subject. The late John Bell was not aware of this fact, although it was he who first discovered and pointed out the true nature of the nævus maternus, and gave the disease the name of Aneurism by anastomosis." 108.

In speaking of the preservation of diseased lung, Mr. Knox makes an observation or two worth recollecting.

"The immersion in alcohol has, it will be particularly observed, a much more powerful effect in corrugating one texture (the cellular) entering into the composition of the organ than another. In all cases the disease should be carefully pointed out by means of bristles, &c. previous to finally closing in the preparation, or even putting it amongst alcohol. I have succeeded in preserving the appearance in apoplexy, tubercles, &c. by securing a *thin* slice of the diseased lung on a wax tablet previous to being put into the alcohol; by this means I perceive the sudden corrugation of the cellular parenchyma of the lung is prevented, at all events from contracting the whole section, and the tubercles are brought prominently into view." 110.

Passing over a good many matters, we make one extract more for the edification of the accoucheurs.

"Teachers of midwifery require a very large museum to enable them to fulfil their duties to their pupils in a proper manner. I have known as much as £300. sterling given for a collection of this sort; and when we reflect that no person can say, 'I want a museum to teach midwifery (or any other branch of the profession), I shall have one made within the year,' I do not think that the sum paid was too much. Collections of this kind can only be formed gradually, and require many years' hard labour, and much expense, to render them, as I

have already remarked, fit to be consulted by any one. Casts and wax models, &c. form at present a large part of these collections, but I rather think that they are not exactly so invaluable as they were considered some years ago; I am of opinion, indeed, that the museum of most teachers of midwifery are at present made up with erroneous views; and, at all events, there are many preparations which can be of little real utility to the teaching the sound principles of that branch of our profession. Thus it seems to me that the series of fœtuses, put up without dissection, and forming so large a part of these museums, are mere store for the anatomist. The gravid uterus, as seen in most cases, I consider as so much *treasure* locked up, for the present locked up, in fact, in a double sense: first, by means of the key of the museum; and, secondly, by the walls of the uterus being entire. All specific aberrations in Nature's productions unexamined and undissected, are mere objects of curiosity; and *amusement* should never form a feature in an *anatomical* museum. I remember, in assisting to draw up a catalogue of the Barclayan Museum, I was forcibly struck with the vast number of objects of curiosity contained in the collection. Amongst others, a *glass article* kept the whole party laughing for an hour; and I recollect of a bottle actually containing a riddle, made of wood. The misfortune of these things is, that they detract the attention of the young student from matters which require the deepest reflection." 131.

All this is very sensible, and, indeed, there are no persons who can read this little manual, without deriving both amusement and instruction from it. The author is an enthusiast—a true, genuine, unmixed curator—as lively and as unadulterated as a bottle of his own whiskey. He does not affect to consider his art inferior to any other in the wide world. Out upon the bastard liberality which affects to think that there are pursuits to be compared with our own. People of this namby-pamby style of mind never do any thing good. We would not, for many a bawbee, that a second edition should expunge the pleasant little egotisms of this. They give it all its charm, and detract not one whit from its usefulness.

It will have been evident to every body that we think this a capital work in its way. We positively insist upon surgeons and students buying it directly. A second edition must come out before Christmas. But mind, Messrs. Adam and Charles Black, publishers and men of pith though you be, alter not one line of those delectable anecdotes, and the thousand and one little pleasantries, that make us chuckle as we change the dirty water of the macerating tub. Alter them not, Messrs. Black, or we will lay our critical switch about your ears, you may depend upon it.

